# Recommendations for the clinical interpretation and reporting of copy number gains using gene panel NGS analysis in routine diagnostics

**DOI:** 10.1007/s00428-019-02555-3

**Published:** 2019-03-19

**Authors:** Astrid Eijkelenboom, Bastiaan B. J. Tops, Anke van den Berg, Adrianus J. C. van den Brule, Winand N. M. Dinjens, Hendrikus J. Dubbink, Arja ter Elst, Willemina R. R. Geurts-Giele, Patricia J. T. A. Groenen, Floris H. Groenendijk, Daniëlle A. M. Heideman, Manon M. H. Huibers, Cornelis J. J. Huijsmans, Judith W. M. Jeuken, Léon C. van Kempen, Esther Korpershoek, Leonie I. Kroeze, Wendy W. J. de Leng, Carel J. M. van Noesel, Ernst-Jan M. Speel, Maartje J. Vogel, Tom van Wezel, Petra M. Nederlof, Ed Schuuring, Marjolijn J. L. Ligtenberg

**Affiliations:** 1 Department of Pathology, Radboud university medical center, Nijmegen, The Netherlands; 2 Princess Máxima Center for Pediatric Oncology, Utrecht, The Netherlands; 3 Department of Pathology, University of Groningen, University Medical Center Groningen, Groningen, The Netherlands; 4 Pathology-DNA, Location Jeroen Bosch Hospital, Den-Bosch, The Netherlands; 5 Department of Pathology, Erasmus MC Cancer Institute, University Medical Center Rotterdam, Rotterdam, The Netherlands; 6 Department of Pathology, Amsterdam UMC, Vrije Universiteit Amsterdam, Pathology, Cancer Center Amsterdam, Amsterdam, The Netherlands; 7 Department of Pathology, University Medical Center Utrecht, Utrecht, the Netherlands; 8 Department of Pathology, PAMM, Eindhoven, The Netherlands; 9 Department of Pathology, Amsterdam UMC, University of Amsterdam, Amsterdam, The Netherlands; 10 Department of Pathology, Maastricht University Medical Center, Maastricht, The Netherlands; 11 Department of Pathology, Netherlands Cancer Institute, Amsterdam, The Netherlands; 12 Department of Pathology, Leiden University Medical Center, Leiden, The Netherlands; 13 Department of Human Genetics, Radboud university medical center, Nijmegen, The Netherlands

**Keywords:** Copy number gain, Amplification, NGS, Targeted therapy, Routine diagnostics, Molecular pathology

## Abstract

Next-generation sequencing (NGS) panel analysis on DNA from formalin-fixed paraffin-embedded (FFPE) tissue is increasingly used to also identify actionable copy number gains (gene amplifications) in addition to sequence variants. While guidelines for the reporting of sequence variants are available, guidance with respect to reporting copy number gains from gene-panel NGS data is limited. Here, we report on Dutch consensus recommendations obtained in the context of the national Predictive Analysis for THerapy (PATH) project, which aims to optimize and harmonize routine diagnostics in molecular pathology. We briefly discuss two common approaches to detect gene copy number gains from NGS data, i.e., the relative coverage and B-allele frequencies. In addition, we provide recommendations for reporting gene copy gains for clinical purposes. In addition to general QC metrics associated with NGS in routine diagnostics, it is recommended to include clinically relevant quantitative parameters of copy number gains in the clinical report, such as (i) relative coverage and estimated copy numbers in neoplastic cells, (ii) statistical scores to show significance (e.g., *z*-scores), and (iii) the sensitivity of the assay and restrictions of NGS-based detection of copy number gains. Collectively, this information can guide clinical and analytical decisions such as the reliable detection of high-level gene amplifications and the requirement for additional in situ assays in case of borderline results or limited sensitivity.

**Electronic supplementary material:**

The online version of this article (10.1007/s00428-019-02555-3) contains supplementary material, which is available to authorized users.

Diagnostic NGS gene panels allow parallel detection of high-level gene amplifications associated with targeted therapy. In the Netherlands, eight molecular pathology laboratories currently have included copy number analyses in their routine NGS work-up and one laboratory is in the midst of the validation procedure (Supplementary Table 1) and [1–3]. Due to the lack of standard procedures and guidelines, the method of determining, interpreting, and reporting these potential clinically actionable gains vary among the different laboratories (Supplementary Table 1). Guidelines to report the clinically relevant sequence variants in cancer (i.e., small indels and single-nucleotide variants) are extensive [4, 5], but are scarce with respect to somatic copy number gains [6]. The Predictive Analysis for THerapy (PATH) project is a national initiative to optimize and harmonize the routine diagnostics in molecular pathology across the Netherlands. A national meeting was arranged, resulting in recommendations for the interpretation and reporting of copy number gains using gene panel NGS data and to support adequate interpretation and clinical decision-making. Here we focus on high-level copy number gains, also described as high-level gene amplifications, that can entail therapeutically targetable aberrations (reviewed in [7]). High-level copy number gains can be more reliably detected than copy number losses or low-level copy number gains when applying gene (hot spot) panels of limited size.

## Detection of copy number gains from gene panel NGS data

With an increased number of genomic DNA (gDNA) template molecules available for targeted sequencing, gene copy number gains will be reflected by an increased abundance of these genomic segments in the libraries and consequently result in an increased number of sequencing reads covering the respective (part of a) gene. To identify these coverage outliers in gene panel NGS data, several approaches have been described [8–11]. For details on methods and software used in routine diagnostics in the Netherlands, see Supplementary Table 1**.** Note that at this moment “best practices” are yet to be determined and the choice of method and software is generally based on in house availability and validation.

**Fig. 1:**
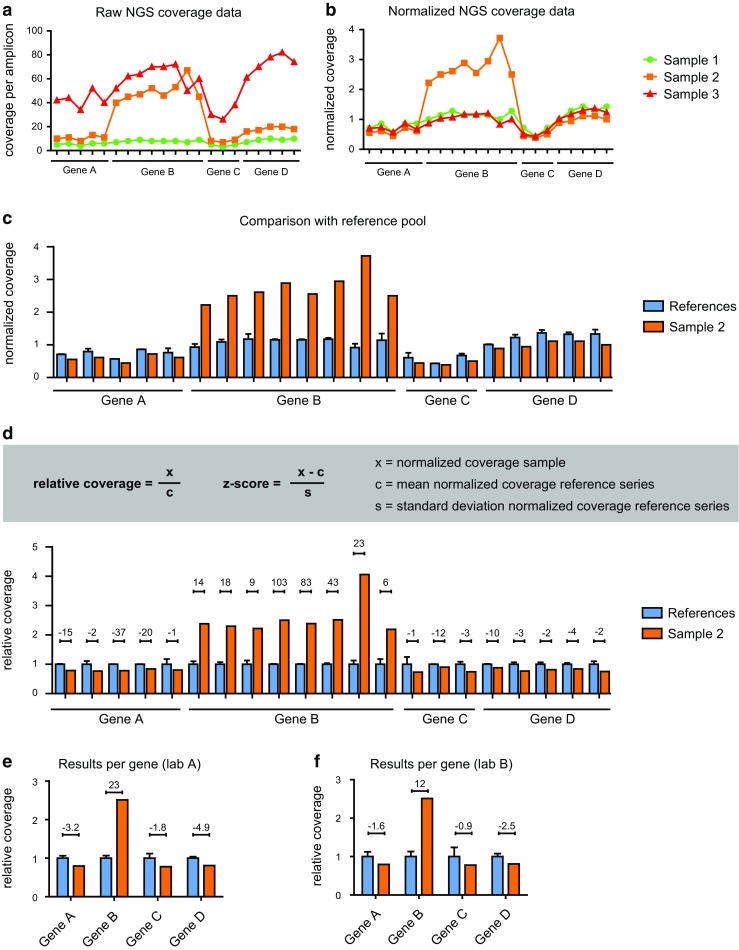
Detection of copy number gains from gene panel NGS coverage data. **a** The absolute coverage per amplicon from theoretical gene panel NGS data for three samples is shown, in which *Gene B* is amplified in sample 2. Multiple data points (i.e., amplicons) are presented per gene. **b** In this example, the normalized coverage per amplicon is obtained by correction with the median coverage of all amplicons within that sample. **c** The normalized coverage allows a comparison with the average normalized coverage of multiple samples in an internal or external reference pool. **d**, **e** Relative coverage (also referred to as “fold-change”) and *z*-scores (depicted above the bars) can be presented per amplicon (**d**) and per gene (**e**). **f** The influence on technical variations is illustrated by results in which the same relative coverage is obtained, with 2-fold increased standard deviations mimicking inter-laboratory technical differences

Generally, these approaches start with sample normalization to correct for differences in total reads, which is especially required in the context of formalin-fixed paraffin-embedded (FFPE) tissue analyses with variable input quality and quantity of gDNA. It is of importance that the applied normalization method is not affected by the presence of high-level copy number gains. For example, the median coverage is more stable relative to average or summed coverage in the presence of high-level copy number gains. In addition, the genomic locations covered by the gene panel should be sufficiently spread throughout the genome covering multiple loci, to allow appropriate normalization in the presence of a high-level amplified gene. Subsequently, the obtained normalized read count per amplicon and/or per gene can be compared to a reference pool of (control) samples to estimate the copy number. From this comparison, statistical measures such as relative coverage (also often referred to as “fold change”) and *z*-scores can be deduced (Fig. 1). The choice for an internal or external reference pool (that is, reference samples are analyzed within the same or a different batch) and the minimal size of this reference pool depends on diagnostic batch size, batch content, and the stability of the assay and should therefore be determined during validation. One should keep in mind that aneuploidy in tumors can affect normalization and the quantification might be an underestimation or overestimation depending on the nature and extend of aneuploidy and the number of genomic regions that are included in the gene panel. Therefore, the gene panel should also include genes/loci that are not expected to be affected by CNV in the malignancies of interest.

The *z*-score is a significance score commonly used in the detection of copy number variation from coverage data. This score represents the number of standard deviations that the obtained coverage is above or below the mean of a reference group of values. Therefore, it greatly depends on testing conditions, including the number of data points for a given locus and the analytical noise that causes variations in control samples or between duplicate analyses of the same sample. In a similar manner, the confidence interval is a statistical measure representing the variation in a reference group of samples and reflects whether the measurement likely lies within the reference group interval. Consequently, while *z*-scores and other measures like confidence intervals are essential to distinguish analytical noise from actual copy number variation and thus reflect the statistical significance of a copy number variation, they are also greatly influenced by the standard deviation that depends on laboratory- and test-specific conditions (see also Fig. 1e, f). In the following paragraphs, the *z*-score is used as a representative for any significance score that can be used to distinguish noise from actual copy number gains.

By applying validated thresholds, significant coverage gains can be identified that reflect gene amplifications. Note that the relative coverage of individual amplicons within an amplified gene can differ. Generally, poorly performing amplicons with a low absolute coverage in control samples tend to obtain a lower relative coverage in case of high-level amplifications, likely due to technical saturation. The required total number of amplicons per gene depends on the technical variability and should be determined during validation. Distribution of amplicons throughout the gene locus is preferred to prevent false positive calls from partial gene amplifications, while keeping in mind frequently deleted regions like the exons 2 to 7 in EGFRvIII [12].

**Fig. 2:**
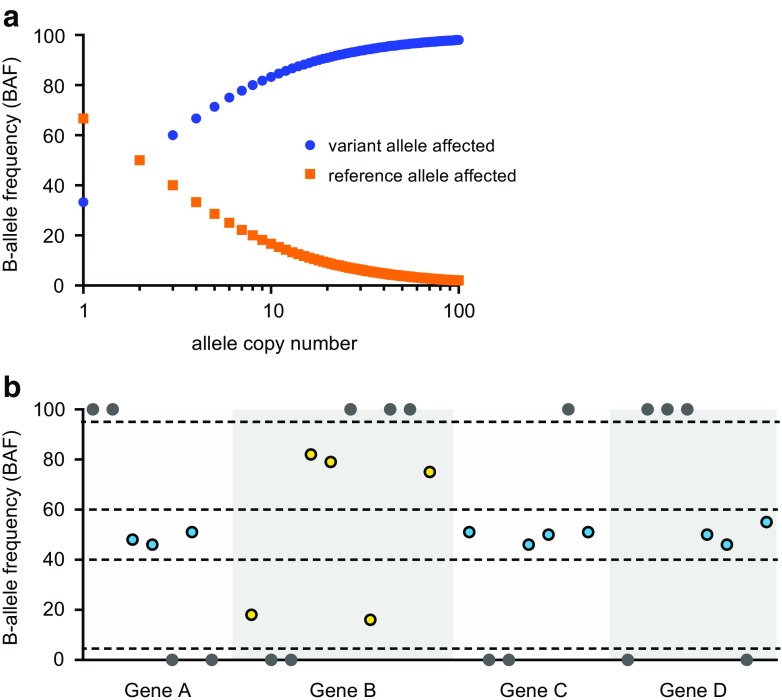
Detection of copy number variation using B-allele frequencies (BAF). **a** In case of heterozygosity, variant allele frequencies (or BAFs) are influenced by copy number variation at the respective loci. Here, for a hypothetical case with a neoplastic cell load of 50% the NGS-based BAF (*y* axis) is shown for an increasing number of alleles (*x* axis). **b** An example of BAFs of common SNPs at the gene loci of the NGS results of sample 2, presented in Fig. 1, in which *Gene B* is amplified. Every circle represents the variant allele frequency of a common SNP. Dark gray circles represent homozygous alleles. Blue circles represent heterozygous alleles for which the BAF is within the expected ~50% (40–60% range). Yellow circles represent heterozygous alleles for which the BAF is divergent from this range due to amplification of the reference allele (decreased BAF) or amplification of the variant allele (increased BAF)

A second commonly used approach to detect copy number variation using NGS data is based on variant allele frequencies of germline (single-nucleotide) polymorphisms (SNPs) at the gene loci. These so-called B allele frequencies (BAFs) were initially described for SNP-based array analysis [13]. For heterozygous SNPs, the variant allele frequency in gDNA from normal tissue approaches 50%. In the presence of copy number gains, the variant allele frequency is increased when the B/minor allele is amplified and likewise decreased with amplification of the A/major allele (Fig. 2).

**Fig. 3:**
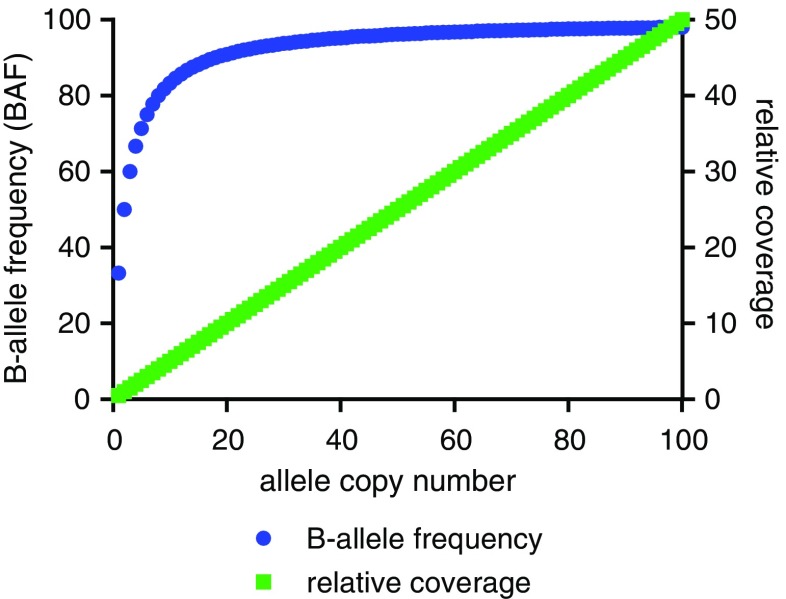
Changes in BAF and relative coverage are affected by the allele copy number. The effect of the number of alleles present in the neoplastic cells on BAF (blue) or relative coverage (green) in case of a neoplastic cell load of 50%. Generally, the BAF values are more divergent with lower number gains like duplications and the resolution decreases with higher-level copy number gains, while relative coverage increases linearly (until technical saturation is reached)

The relative coverage and BAF approaches are complementary. For example, the BAF approach requires coverage information to discriminate copy number gains from copy number losses. With sufficient “SNP-density” the BAF approach can be more sensitive to detect low copy number aberrations (such as gene deletions or duplications), while the relative coverage approach is more reliable in the quantitative assessment of higher-level copy number gains (Fig. 3).

Regardless of the applied method, it is recommended to use positive and negative control samples in which the copy number gains are confirmed by alternative approaches such as fluorescence in situ hybridization (FISH), SNP-array analysis, or multiplex ligation-dependent probe amplification (MLPA). After validation, positive and negative control samples should also be analyzed on a regular basis, to ensure stability of the assay. Analytical cutoff values should be established that translate into reliable and significant copy number gains, preferably for all individual genes of interest. Since analysis of gDNA of limited input quantity and/or quality may result in suboptimal coverage and subsequently lead to false positive calls, the use of minimal coverage thresholds is also recommended.

## Clinically relevant measures of gene amplification

**Fig. 4:**
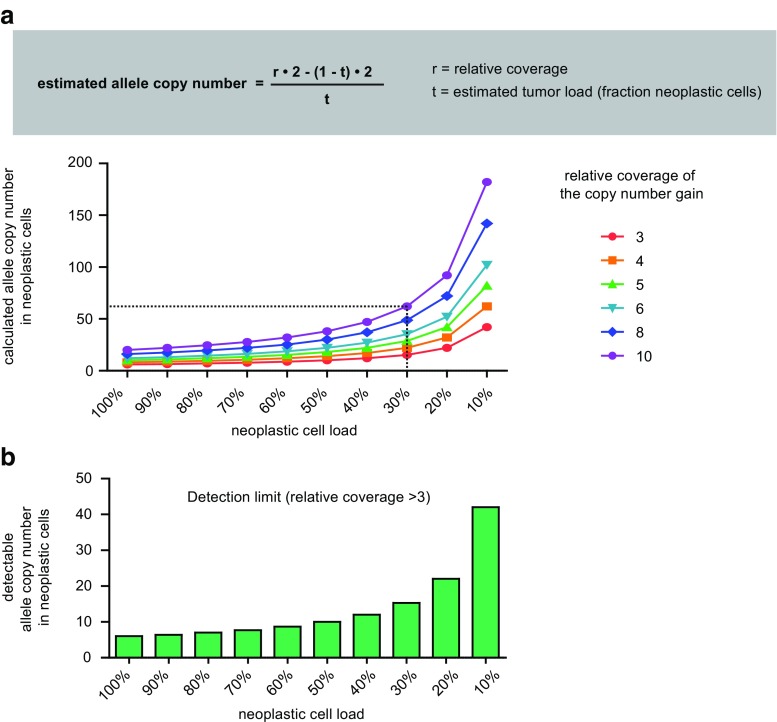
The relative coverage is affected by both neoplastic cell load and the allele copy number of the amplified gene. **a** The allele copy number can be estimated from relative coverage. Here, the copy number (*y* axis) is calculated with decreasing neoplastic cell load (*x* axis) for a range of relative coverages. For example, a relative coverage of 10 in case of a neoplastic cell load of 30% represents an estimated copy number of 62 alleles (see dashed line). **b** The detection limit of the assay can be estimated based on neoplastic cell load. In this example with a relative coverage of 3.0 as a validated analytical cutoff, the minimum detectable allele copy number in the neoplastic cells is shown with decreasing neoplastic cell load

Currently, the clinical relevance of gene amplifications is largely based on molecular analyses by in situ approaches such as FISH. The presence of gene copy number gains in single neoplastic nuclei has been correlated with clinical responses towards drugs targeting the product of the amplified gene. However, the above-described, NGS-based measurements are obtained from the total gDNA template molecules in the sample and as such represent a mixture of tumor-derived and non-neoplastic gDNA from stromal and inflammatory cells. The measured gain is thus determined by both the neoplastic cell percentage and the actual allele copy number (Fig. 4a). To relate the NGS detected gains to FISH detected gains, the calculated number of alleles could be corrected for the estimated percentage of neoplastic nuclei in the area from which the gDNA was isolated. While we realize the estimation of this percentage is error-prone [14, 15], it can be supported by the variant allele frequencies (VAF) of somatic variants in other genes and it allows estimation of the number of gene copies in the “order of magnitude” required to asses clinical relevance. As mentioned above, the estimation of the actual copy number gains may be biased in highly aneuploid tumors. For clinical decision making, high copy number gains are most relevant for which the estimation of allele copy number will be less affected compared to low copy number alterations.

It is important to assess the clinical relevance of an estimated copy number on a case-by-case basis. For example, for the tyrosine kinase inhibitor (TKI) crizotinib a FISH established cutoff of 10 copies of the *MET* gene in lung adenocarcinoma has been suggested [16], while only very limited data on the response related to low-level (4–9 copies) and high-level (≥ 10 copies) amplification are presently available [17, 18]. For capmatinib targeting *MET* in *EGFR* mutated lung adenocarcinoma with disease progression on EGFR-TKI treatment, gains of ≥ 6 copies have been related to response to combined *MET* and *EGFR* targeting [19]. For example, in case an estimated copy number gain of 30 copies of *MET* in the tumor cells is detected by gene panel NGS analysis, TKI treatment could directly be considered, while an estimated number more closely to 6 copies warrants subsequent FISH analysis for clinical guidance. Depending on the results of the internal validation, a confirmatory FISH analysis can be performed in a subset of cases. It is important to realize that these cutoffs differ per therapeutic agent, gene, and tumor type. To determine the optimal cutoff for treatment responses, several trials are ongoing; e.g., *FGFR1* amplification levels predicting clinical benefit for inhibitors targeting FGFR1 appear drug and tumor dependent [20].

Note that in FISH analyses typically only 50–100 selected nuclei are analyzed. While this enables specific investigation of neopastlic nuclei, which is hampered by admixture of non-neoplastic cells in NGS-based analysis, it creates a potential bias that this selection is not representative for the whole neoplastic area (for instance, the selection can favor nuclei with multiple signals). For NGS-based approaches, the data represents an average of a much larger number of cells (e.g., 20 ng represents ~3300 nuclei). To ultimately evaluate the clinical use of FISH- and NGS-based diagnostics, both should be included in clinical trials focusing on treatment response.

## Clinical reporting of NGS-based copy number gain analysis

The clinical report that describes the results of the NGS-based analysis of copy number gains should include the interpretation, e.g., “amplification detected of *gene X*,” “no indications for the presence of amplifications,” “inconclusive,” or “additional testing required.” In addition, it is essential to include quantitative information as well as assay limitations.

Quantitative information includes the relative coverage that reflects the total number of alleles present in the gDNA sample. Relative to BAFs, this measure provides a more linear read-out of allele copy number. For loci with a significant copy number change, based on the minimal validated relative coverage and *z*-score or confidence interval, it is recommended to report the relative coverage and the estimated number of copies in the neoplastic cells by taking the estimated neoplastic cell percentage into account.

Awareness of assay limitations is critical for routine diagnostics, as illustrated by the necessity to include the “reportable range” for NGS-based detection of sequence variants, like the fraction of the targeted genomic regions for which calls of an acceptable quality can be generated [5]. For the report of copy number gain detection, it is essential to specify the estimated sensitivity as the minimum amount of gene copies that can reliably be measured as a copy number gain. Note that generally these NGS-based analyses are not sufficiently sensitive to reliably exclude the presence of any copy number gain in case of low neoplastic cell percentages. Therefore, the threshold for copy number gains also depends on the percentage of neoplastic cells (Fig. 4b). We suggest a sentence such as “Based on the estimated neoplastic cell percentage, this assay allows detection of copy number gains of > XX copies.” This sensitivity is also crucial to decide on the necessity to perform additional analyses.

## Conclusions

High-level copy number gains (gene amplifications) of clinically targetable genes can be detected using NGS gene (hot spot) panels in which multiple independent genomic regions are included. Tables 1 and 2 summarize the technical considerations and biological phenomena impacting on the detection of these copy number gains. NGS gene panel-based analysis has the advantage that high-level copy number gains in multiple genes can be measured simultaneously in addition to sequence variant detection. As such, independent assays to determine copy number gains may not be needed for a subset of tumors, improving turn-around times, cost-efficiency, and tissue management. A limitation of an NGS-based approach is the difficulty to detect low-level copy number gains and/or high-level amplifications in specimens with low neoplastic cell percentages. For these cases, in situ analyses including FISH are recommended to either exclude or confirm the presence of copy number gains. We recommend reporting relative coverage and the estimated copy numbers in neoplastic cells for loci that reach a minimal validated relative coverage and significance score, as these parameters represent both quantitative and clinically relevant measures. Clinical validity of the identified copy number gain needs to be interpreted on a case-by-case basis.

**Table 1:** Technical considerations for detection of copy number gains (gene amplifications) using panel NGS data

Technical issue	Why relevant?	Considerations
Panel content	Panel size and selection of genomic loci can affect detection of copy number gains	(i) Contains amplicons/probes sufficiently spread throughout the genome (ii) Includes loci likely to not be affected by copy number variation in tumor of interest (iii) Minimal number of amplicons/probes per gene, preferably throughout gene locus (iv) For BAF, include sufficient number of heterogeneous loci for sufficient “SNP-density”
Normalization	Required to correct for differences in gDNA input quality/quantity	Choose method that is not/minimally affected by copy number variation
Reference pool	Is required to detect coverage outliers indicative of copy number gains	(i) Internal and/or external reference pool (ii) Includes samples without copy number variation (e.g., normal tissue) (iii) Processed using identical protocols
Thresholds	Required to distinguish genuine copy number gains from technical noise	(i) Validated by positive/negative controls using other methods (ii) Includes minimal coverage thresholds to prevent false positive calls from poor quality gDNA (iii) Include positive and negative controls on a regular basis, to ensure assay stability and test validated thresholds
Sensitivity	Awareness of assay limitations is critical for routine diagnostics	(i) Affected by thresholds and neoplastic cell percentage (ii) Should be included in clinical report

**Table 2:** Biological phenomena that affect the detection of copy number gains using panel NGS data

Biological phenomena	Why relevant?	How does it affect detection of copy number gains?
Neoplastic cell content	Measurements are obtained from a mixture of tumor-derived and non-neoplastic gDNA	(i) The actual detected increase in coverage/deviation in BAF increases with neoplastic cell content (ii) Influences the estimation of the allele copy number (iii) Determines assay sensitivity (in combination with thresholds used to identify statistically significant gains)
Allele copy number/magnitude of amplification	Clinical consequences are based on cutoffs in allele copy number of gene amplification	(i) The detected increase in coverage/deviation in BAF increases with allele copy number (ii) Assay sensitivity should match the clinically relevant cutoffs in alllele copy number
Aneuploidy	Can affect normalization and allele copy number estimation	(i) Results in underestimation or overestimation depending on the nature and extend of aneuploidy and the number of genomic regions that are included in the gene panel (ii) High-level copy number gains are likely less affected compared to low copy number alterations

## Electronic supplementary material

Supplementary Table 1 An overview of the approaches for copy number analyses in routine gene panel NGS using for predictive testing of FFPE specimen work-up of nine hospital-based molecular pathology laboratories in the Netherlands (April 2018). (XLSX 12 kb)
